# Supplementary material for: Predictors of neonatal mortality among neonates in Tigray regional state, Ethiopia: A cross-sectional study
Source: PLoS One. 2024 Dec 17;19(12):e0315400. doi: 10.1371/journal.pone.0315400 (PMC11651602; doi:10.1371/journal.pone.0315400)
Supplement: Attachment — Submitted filename: Response to the reviewers - 1.pdf [file pone.0315400.s002.pdf]

To: Editor-in-Chief

PLOS ONE

Date: 02-OCT-2024

Dear Editor-in-Chief,

First of all, we would like to thank the editors and reviewers who worked on our paper for their considerable efforts and for the time they devoted to our work. Our paper entitled *"**Predictors of neonatal mortality among neonates in Tigray regional state, Ethiopia: a retrospective cross-sectional study**"* is being published in your esteemed journal. We have been attaching the revised manuscript of the paper prepared by taking all the constructive comments and suggestions of the respected referees into consideration. I strongly hope and believe that you will find the revised version of the manuscript suitable for publication in your esteemed journal, ***PLOS ONE***. Point-by-point, the authors' responses to the referees comments and suggestions are given in the following for your consideration and to require action in this regard. Looking forward to receiving an earliest communication in this regard!

Thank you so much,

With warm regards,

Gebru Gebremeskel Gebrerufael.

Department of Statistics, College of Natural and Computational Science, Adigrat University, Ethiopia

On the behalf of the other authors

Authors' response to the reviewers' comments

Ref: Submission ID PONE-D-24-30270

Title: Predictors of neonatal mortality among neonates in Tigray regional state, Ethiopia: a retrospective cross-sectional study

Authors:

Gebru Gebremeskel Gebrerufael (gebrugebremeskel2015@gmail.com)

| **ORID:** https://orcid.org/0000-0003-2795-8529

Brhane Gebrehiwot Welegebrial (brhanegb008@gmail.com | **ORCID**: https://orcid.org/0000-0003-3600-7477)

Mehari Gebre Teklezgi (meharistat@gmail.com | **ORCID**: https://orcid.org/0000-0002-3638-6086)

Version: 1 Date: 19-Sep-2024

We would like to thank you so much the respected editors and reviewers who worked on our paper for their considerable efforts and for the precious time they devoted to our work in order to concise and improve the quality of our manuscript. We have made almost all the changes requested and enclose a revised version of the manuscript (changes to our paper can be highlighted **in purple** in the main document). Our point-by-point authors' responses to comments received in review are summarized in this document, inserted **in purple,** and given in the following below. Moreover, we strongly hope that the quality of the revised manuscript has been enormously improved after taking the respected reviewers' constructive comments and suggestions into consideration.

Editors Comments:

Reviewer #1

This manuscript titled "Predictors of Neonatal Mortality among Neonates in Tigray Regional State, Ethiopia: A Retrospective Cross-sectional Study" focuses on neonatal mortality, a critical concern, especially in low-resource settings such as Ethiopia. The use of secondary data from the 2016 Ethiopia Demographic and Health Survey provides a **solid foundation for identifying** risk factors, and the application of multivariable logistic regression for data analysis is appropriate for the study objectives. The study contributes valuable insights to the existing body of knowledge regarding neonatal mortality at the regional level in Tigray and its predictors.

Constructive feedback:

Comment 1: L71: "As a result, this report shows that it has continued to perform at high level." This statement is incongruent with the rest of the paragraph and does not specify what the performance is about or whose performance it is referring to.

Response 1: Thank you very much**.** The authors highly appreciated your insightful, constructive suggestions. Therefore, the authors have modified the manuscript according to your suggestions.

Comment 2: Study design: In this study, the authors used secondary data from the DHS, there is no temporarily neither in data collection nor in participant enrolment. Even the DHS data collection was at one point in time. Therefore, the term "retrospective" does not fit into the study design. It is a cross-sectional study by analysis of secondary data.

Response 2: Thank you very much**.** The authors highly appreciated your insightful, constructive suggestions. Therefore, the authors have removed it the word retrospective overall the manuscript according to your suggestions.

Comment 3: Figure 1: Do not use comas to separate figures of a number when it is not up to a thousand.

Response 3: Thank you a lot**.** We authors removed it the commas from figure 1. See on **Figure 1**

Comment 4: L123: Do not begin a sentence with figures

Response 4: Thanks so much. The authors have corrected it based on your suggestions. See on page #05 and line#120-121.

Comment 5: L124: "have all been questioned since the 2016 EDHS." This is confusing. Has there been additional questioning of these families after the DHS? What was the purpose f the additional questioning?

Response 5: Thank you very much, **Reviewer.** The authors highly appreciated your insightful, constructive suggestions. Therefore, the authors have modified the manuscript according to your suggestions.

Comment 6: L126 "Preceding the research." Please be consistent with differentiating between your study and the DHS. Specify whether the word "research" here refers to the DHS or your study.

Response 6: Thank you very much, **Reviewer#1.** The authors highly appreciated your insightful, constructive suggestions. So, the authors have modified according to your suggestions.

Comment 7: L127 The word "locate" insinuates that contact was made with participants. Consider changing to "identified"

Response 7: Thanks so much. The authors have corrected it based on your suggestions. See on page #05 and line#122.

Comment 8: Your methodology does not clearly describe inclusion and exclusion criteria for the

DHS nor those for your study selection. You do mention including cases with complete records but you have not specified which records (variables) you were interested in from the DHS.

Response 8: Thank you very much, **Reviewer.** The authors highly appreciated your insightful, constructive suggestions. Therefore, in this study total number of neonates whose mothers asked for the interview and those whose age was below one month. Moreover, those children's whose dependent variables have missing are excluded from the study. Therefore, those children whose age greater than one month, whose outcome variable missing and also those children whose regions out of Tigray region from the EDHS were excluded from this study.

Comment 9: L143 The description of neonatal death is incorrect. Aparently only deaths in the first week of life and pregnancy losses after 4 weeks of gestation were considered. This is not consistent with the usual estimation of neonatal mortality rates which includes deaths in the first 28 days of life and excludes pregnancy losses.

Response 9: Thank you very much, **Reviewer.** The authors highly appreciated your insightful, constructive suggestions. Therefore, the authors have removed these words and modified it according to your suggestions. See on the method section.

Comment 10: L199 The highest proportion of neonatal deaths, not death rates

Response 10: Thanks so much. The authors understand as type of category variable divided by the sum of alive and died child's. For example proportion of death for 35 and above =

$$\frac{[n(35\text{ and above })]*100}{[n(Alive\ for\ 35\text{ and above })+n(Died\ for\ 35\text{ and above })]}$$

Comment 11: L202 "in a similar neonatal…." This phrase is unclear

Response 11: Thanks so much Reviewer#1. The authors have corrected it based on your productive suggestion. See on the Result Section page#09 and line#209.

Comment 12: L202: “moms of multiple birth types” Do you mean moms who had multiple babies from the same pregnancy or moms who had several types of birth?

Response 12: Thanks so much **Reviewer#1.** The authors mean that “mothers who had multiple babies from the same pregnancy”. See on the Result Section page#08 and line#199-200.

Comment 13: L255 change Cameron to Cameroon

Response 13: Thanks a lot **Reviewer#1**. The authors have corrected it based on your productive suggestion. See on the Discussion section page#10 and line#245.

Comment 14: In the discussion section, add a paragraph on how the study findings can be useful to policy makers in the strife to reduce neonatal mortality rates, with possible examples of strategies that have been applied in other settings where similar predictors were found.

Response 14: Thanks a lot **Reviewer#1**. The authors have amended it according your suggestion. See on the Discussion section page#11 and line#277.

Comment 15: Add a section on limitations of the study.

Response 15: Thank you very much, **Reviewer.** The authors highly appreciated your insightful, constructive suggestions. Therefore, the authors have added according to your suggestions. See on the Limitation of the study section above Conclusion.

Comment 16: L279 You earlier mention that Ethiopia achieved MDG with reduction of U-5 mortality, but neonatal mortality rate increased (L70). Here you are making a contradicting statement.

Response 16: Thank you a lot**.** The authors wants to say that “mortality rate of under five children have reduction”. However, the neonatal mortality rate could not achieve it. Then we authors modified it according your suggestion.

Comment 17: L97 – 104 Write out your objective and specific objectives in a paragraph format, not bullet points.

Response 17: Thanks so much. The authors have corrected it based on your productive suggestion. See on the introduction section.

Comment 18: L211-217 This description of methods of analysis should be moved to the methodology section of the manuscript.

Response 18: Thank you very much, **Reviewer.** The authors highly appreciated your insightful, constructive suggestions. See on **Method section** page 07 and line number 165-170.

Comment 19: The authors report adjusted odd ratios but do not tell what was adjusted for.

Response 19: Thanks a lot **Reviewer#1**. The authors mean that AOR analysis of overall predictor variable with dependent variable. Not one predictor with dependent variable that means that COR.

Reviewer #2:

Abstract section

Comment 1: The sentence the predictors aren't well recognized could be rephrased to be more formal and precise. A clearer version could be: **the predictors are not well understood**.

Response 1: Thank you a lot **reviwer#2** for suggestion. The author's couldn't found the specific sentences. Pease put the page number and line number of this manuscript in order to give a clear justification and made an amendment.

Comment 2: Neonates born to the mother's age (20–34 years) – this phrase is slightly confusing. It would be clearer to say: neonates born to mothers aged 20–34 years

Response 2: Thank you so much **Reviewer #2.** We really appreciate for your constructive comments. We have authors corrected according your suggestions.

Comment 3:"Tigray regional state was experiencing a higher neonatal mortality rate than the national level" could be rephrased for clarity: The neonatal mortality rate in Tigray regional state was higher than the national average.

Response 3: Thank you very much**.** The authors highly appreciated your insightful, constructive suggestions. Therefore, the authors have corrected according to your suggestions.

Comment 4: The conclusion section could be more impactful by briefly emphasizing the implications of the findings. For example: "To reduce neonatal mortality, targeted interventions should focus on high-risk groups, such as mothers delivering at home and those with multiple births."

Response 4: Thank you very much**.** The authors highly appreciated your insightful, constructive suggestions. Therefore, the authors have corrected according to your suggestions.

Comment 5: The sentences "male neonates were more likely to die" is somewhat abrupt and

could be softened for tone. Consider: “Male neonates had a higher risk of mortality compared to females.”

Response 5: Thank you very much**.** The authors highly appreciated your insightful, constructive suggestions. Therefore, the authors have corrected according to your suggestions. See on Result section page #10-11 and line #256-259.

Introduction section:

Comment 6: The sentence “The probability that a neonate would die within the first 28 days of life, or the first four weeks of life, is known as the neonatal mortality rate (NMR)” is a bit lengthy and could be streamlined for clarity: “Neonatal mortality rate (NMR) refers to the probability of a neonate dying within the first 28 days of life.”

Response 6: Thanks so much. The authors have corrected it based on your productive suggestion. See on the **Introduction section** page #02 and line #40-41.

Repetitiveness:

Comment 7: There are some repetitive statements about neonatal mortality, particularly in the first few paragraphs. For instance, the information about the percentage of child deaths due to neonatal mortality is mentioned multiple times. You could combine these points for better readability.

Response 7: Thanks a lot reviewer. The authors have minimized according your suggestions. See on the Result section.

Missing Gap Explanation:

Comment 8: While the introduction explains the high neonatal mortality rate in Tigray and Ethiopia, it could better emphasize the specific research gap that this study aims to fill. For example, you could more explicitly state: “Despite various studies on neonatal mortality in

Ethiopia, research focused specifically on regional variations, especially in the Tigray region, is limited.” This would highlight the study's novelty.

Response 8: Thanks so much. The authors have corrected it based on your productive suggestion. See on the **Introduction section** page #03 and line #74-75.

Transition to Objectives:

Comment 9: The transition from the background information to the study objectives is abrupt. Adding a sentence that ties the context to the specific aims of the study would improve the flow. For instance: “Understanding these risk factors in the context of Tigray can provide targeted interventions and inform public health strategies.”

Response 9: Thanks so much. The authors have modified on some parts of the introduction based on your productive suggestion. See on the **Introduction section.**

Minor Stylistic Adjustments:

Comment 10: In the sentence “the neonatal mortality rate (NMR) accounts for about 44% of deaths in children under five”, it would be clearer to say: “Neonatal mortality accounts for approximately 44% of deaths in children under five.”

Response 10: Thank you a lot **reviwer#2**. The authors have corrected it based on your productive suggestion. See on the **Introduction section** page #02 and line #44-45.

Method section:

Sample Size and Population

Comment 11: The methods section mentions that 716 neonates were included in the analysis. However, the justification for why this sample size was selected and whether it is statistically sufficient for identifying risk factors is not clearly addressed. Adding a brief explanation of sample size determination or its adequacy would enhance clarity.

Response 11: Thanks so much for your suggestion. However, in Tigray regional state there was 4428 total number of live births of under five children and out of 716 Childs whose who have completed records. For further clarification see Figure 1 on the Diagrammatic presentation of sample selection.

Lack of Explanation on Missing Data:

Comment 12: It's unclear how missing data were handled. Since secondary datasets often have incomplete data, it would be important to state if any neonates were excluded due to missing information or how missing data were managed (e.g., imputation methods, exclusion).

Response 12: Thanks so much. The authors have excluded those incomplete values of the response variables. See Figure 1 on the Diagrammatic presentation of sample selection.

Ethics Considerations:

Comment 12: Although it is mentioned that ethical approval was not required due to the use of publicly available data, this section could be expanded to clarify how data confidentiality and anonymity were maintained.

Response 12: Thanks a lot **Reviewers#2.** Permission was obtained from the DHS and the names of participants were not registered in the questionnaire and their unique numbers were kept locked for better confidentiality.

Data Analysis Section Could Be Expanded:

Comment 13: While the use of binary logistic regression is mentioned, there is little detail about the selection of covariates or how multicollinearity was checked. It would strengthen the methodology to explain how variables were chosen for the model and whether tests for multicollinearity or interactions between variables were conducted.

Response 13: Thanks a lot **Reviewers#2** for your suggestion. In the bi-variable logistic regression model analysis was done for each independent variable with the outcome variable and all predictor variables were included in the multivariable logistic regression model analysis to identify predictor factors of neonatal mortality rate. We authors have begun here first by checking the multicollinearity test was done to assess the existence of correlation among the predictor variables. Since the VIF value for all predictor variables is < 10 there is no multicollinearity problem (if necessary the author will be attached as supplementary materials). And also, there is no interaction effect between the covariate variables.

Flow of Methods:

Comment 14: The flow of the methods section can be improved by reorganizing the sections more logically. For instance, after explaining the study design, data source, and sample size, the section should discuss variables and their definitions, followed by the statistical analysis plan. The current flow jumps between these topics, which can be confusing.

Suggestion: Consider this sequence for the methods:

1. Study design and data source.
2. Sample size and population.
3. Definitions of dependent and independent variables.
4. Data analysis (descriptive and regression models).
5. Ethical considerations.

Response 14: Thank you very much **Reviewer #2**. The authors amended according your suggestion. See on Method section.

Comment 15: Results section:

Overuse of Numbers:

In the narrative portion of the results, there is an overemphasis on reporting numbers and percentages without sufficient explanation. While numbers are important, the results section could benefit from more interpretation and context around these findings.

Interpretation of Tables: While tables are provided, there is minimal interpretation of the data in the narrative. The text mainly repeats the numbers found in the tables. More effort could be made to explain the patterns or trends observed in the data.

Response 15: Thanks so much. The authors amended some parts of the result section according your suggestion. See on Result section.

Comment 16:

Goodness-of-Fit Test:

The results briefly mention that the Likelihood Ratio Test (LRT) and Hosmer-Lemeshow test were used to assess model fit, but there is little interpretation of what these results mean. This could confuse readers unfamiliar with these tests.

Response 16: Thanks so much. Thanks so much. The authors have corrected it based on your productive suggestion. See on the Method section.

Comment 17:

Subtle Presentation of Non-Significant Findings:

Some findings, such as ANC follow-up and toilet facility, are reported as non-significant predictors, but these could be better highlighted. Readers might overlook these aspects, which are important in understanding what did not contribute to neonatal mortality.

Response 17: Thank you a lot**.** However, these significant predictors variables may be in our study no-significant may be due to small sample sizes, study design etc.

Comment 18: Discussion section:

Limited Discussion of Non-Significant Findings:

The discussion focuses heavily on significant predictors but does not adequately address the factors that were not significant (e.g., antenatal care (ANC) follow-up, mother's education, and toilet facilities). Discussing these non-significant results could provide important insights, such as why some expected predictors did not show a relationship with neonatal mortality in this region.

Response 18: Thank you a lot for your suggestion**.** We authors have modified it according your suggestion. See on page #11 and line#274-276.

Comment 19: Inconsistent Depth in Comparisons with Other Studies:

While some comparisons with previous studies are well-done, others are underdeveloped. For example, the finding related to birth order could be discussed in more detail, as other studies might have identified different trends.

Response 19: Thank you so much**.** We authors have modified it according your suggestion. See on page #10 and line#247.

Comment 20: Lack of Explanation for Unexpected Findings:

Some findings, like the reduced neonatal mortality risk for mothers aged 20–34, could be explored more thoroughly. The discussion doesn't sufficiently explain why younger mothers (under 20) did not show the expected increased risk of neonatal mortality.

Response 20: Thank you a lot for your comments. Those mothers whose age (20–34) have less neonatal mortality it may be less giving attention because they have another Childs.

Comment 21: Overemphasis on General Findings:

While the discussion covers the main findings well, it sometimes reiterates the results without

adding much interpretation. For example, the finding about male neonates being more likely to die is mentioned without deeper exploration of why this might be the case beyond biological vulnerability.

Response 21: Thank you so much**.** We authors have modified it according your suggestion. See on page #11 and line#258-259.

Comment 22: Recommendations Could Be More Specific:

While the study provides general recommendations, they could be more targeted. For instance, rather than simply suggesting improvements in health services, the discussion could highlight specific interventions like community education on safe delivery practices, promotion of facility-based births, or specialized care for multiple births.

Response 22: Thanks so much. The authors have corrected it based on your productive suggestion. See on the **conclusion section.**

Comment 23: Need for More Regional Comparisons:

Although the discussion compares the findings to national and international data, it could be enhanced by more detailed regional comparisons within Ethiopia. This would help highlight whether the situation in Tigray is unique or reflective of broader trends in the country.

Response 23: Thank you so much**.** We authors have modified it according your suggestion. See on page #10 and line#247.

Comment 24: Limitations Section Could Be Expanded:

The limitations of the study are mentioned but could be more detailed. For instance, the reliance on secondary data from EDHS means there may be important variables that were not captured or measured accurately, such as quality of health services or cultural practices.

Response 24: Thanks so much. The authors have corrected it based on your productive suggestion. See on the limitation of the study section.

Reviewer #3:

General comments: The research is scientifically sound and will significantly contribute to the scientific body of knowledge, particularly in the region of Tigray. However, a few areas need to be improved and proofread. I tracked and changed the document; you can find the comments, suggestions, and changes.

Questions and suggestions

Comment 1: There is 2019 EDHS mini data, and some research has already been published using the data. Why did you not use the recently updated data?

Response 1: Thank you so much **Reviewer #3.** We really appreciate for your constructive comments. We authors have corrected and added in the limitation of the study. See on the limitation of the study.

Comment 2: Background page 4, line 89: A few studies have been conducted in the Amhara, Sidama, and other regions.

Response 2: Thank you so much. We really appreciate for your constructive comments. These conducted studies in Amhara, Sidama, and other regions may not represent for national level of regions. Because these studies taken its sample from specific places/ hospitals only. So, this may not emphasis on the amendment regional policy and interventions.

Comment 3: Method and materials page 5, line 108: Did you conduct a survey? I think you accessed secondary data from the EDHS database.

Response 3: Thank you so much **Reviewer #3**. We really appreciate for your constructive comments. We authors did not say that the survey was conducted us. However, "the survey was

conducted in January and June of 2016 by the Ministry of Health (MOH), the Central Statistics Agency, and the Ethiopian Public Health Institute (EPHI)".

Comment 4: Result section page 10, line 219: Did you calculate relative risk? I thought you used the ODDS ratio to predict factors influencing neonatal death.

Response 4: Thank you so much. We really appreciate for your constructive comments. We authors have corrected it.

Comment 5: Result section page 11, line 231: Where do you calculate 75%? I tried calculating the percentage, but it did not generate this number. Do you consistently use the ODDS ratio to interpret your findings?

Response 5: Thank you so much. We really appreciate for your constructive comments. We authors have corrected it. See on page 09 and line#219.

Comment 6: Result section page 11, line 238: Is this a relative risk?

Response 6: Thank you so much. We really appreciate for your constructive comments. We authors have corrected it.

Comment 7: Result section page 11, line 240: Where do you calculate 88%? I tried calculating the percentage, but it did not generate this number. Do you consistently use the ODDS ratio to interpret your findings?

Response 7: Thank you so much. We really appreciate for your constructive comments. We authors have corrected it. See on page 10 and line#229.

Comment 8: Discussion section, page 12, line 260: How will this variable possibly cause the discrepancy?

Response 8: Thank you so much **Editors**. We really appreciate for your constructive comments." Because these like study's **design, settings, follow-up time, population sample**

size, and sociodemographic of its participants may be increase or decrease the neonatal mortality.

Comment 9: Discussion section page 12, lines 272-274: The possible explanation of the discrepancy of the findings.

Response 10: Thank you a lot. We really appreciate for your constructive comments. This possible discrepancy may be those older age mothers could not attention for caring their Childs.

260 explained **Comment 11:** Conclusion and recommendation section, page 13, lines 287-294: The recommendations are not adequate, especially for identified factors such as home delivery.

Response 11: Thank you so much. We really appreciate for your constructive comments. We authors have corrected it. See on page #12 and line#289-291.
