# Supplementary material for: Predictors of neonatal mortality among neonates in Tigray regional state, Ethiopia: A cross-sectional study
Source: PLoS One. 2024 Dec 17;19(12):e0315400. doi: 10.1371/journal.pone.0315400 (PMC11651602; doi:10.1371/journal.pone.0315400)
Supplement: Attachment — Submitted filename: PONE-D-24-30270_reviewerCommented by Reviewer.docx [file pone.0315400.s001.docx]

PLOS ONE

Predictors of neonatal mortality among neonates in Tigray regional state, Ethiopia: a retrospective cross-sectional study

--Manuscript Draft--

| Manuscript Number: | PONE-D-24-30270 |
| --- | --- |
| Article Type: | Research Article |
| Full Title: | Predictors of neonatal mortality among neonates in Tigray regional state, Ethiopia: a retrospective cross-sectional study |
| Short Title: | Predictors of neonatal mortality among neonates in Tigray regional state, Ethiopia |
| Corresponding Author: | Gebru Gebremeskel Gebrerufael  Adigrat University College of Natural Sciences Adigrat, ETHIOPIA |
| Keywords: | Neonates, Neonatal mortality, Tigray Region State, Logistic regression model |
| Abstract: | Abstract  Background: Since 2015, Ethiopia is committed to lowering the death rate for children under five and it is one of the countries in Sub-Saharan Africa that has accomplished the fourth Millennium Development Goal. However, in Ethiopia, neonatal death has remained a serious public health concern, with greater rates found in Tigray regional state and the predictors aren’t well recognized . The goal of this study was to ascertain the prevalence of neonatal death in the Tigray regional State as well as any relevant risk factors.  Methods: This study performed a secondary data analysis of the 2016 Ethiopia Demographic and Health Survey (EDHS) report . Information was gathered on 716 neonates who were born five years before the survey began. Risk factors for neonatal mortality were thought to include mother and neonate demographics, health, and environmental factors. The study employed multivariable logistic regression model analysis and descriptive statistics to identify significant correlates of neonatal mortality. Results: In Tigray regional state, the overall prevalence of neonatal mortality was 4.3% (95% CI: 3.06, 6.10). The multivariable logistic regression model analysis revealed that factors such as multiple birth types (AOR = 15.3, 95% CI: 3.54, 65.84), birth order (2–4) (AOR = 4.88, 95% CI: 1.52, 15.7), sex of the neonate (being male)  (AOR = 3.75, 95% CI: 1.45, 9.75), home place of delivery (AOR = 7.4, 95% CI: 2.0,  27.6), and neonates born to the mother’s age (20–34 years) (AOR = 0.23, 95% CI: 0.087, 0.58) were significantly risk factors associated with a higher risk of neonatal mortality rate.  Conclusions: The study recognized the sex of the neonate, birth order, mother’s age, place of delivery , and birth type as potential risk factors for neonatal mortality. The prevalence of neonatal mortality indicated that Tigray regional state was experiencing a higher neonatal mortality rate than the national level. To improve services and strengthen the capacity of local stakeholders to address the primary predictor factors that impact neonatal mortality in the nation, the Tigray regional state has to make corrections to its public health strategy and design. |
| Order of Authors: | Gebru Gebremeskel Gebrerufael |
|  | Brhane Gebrehiwot Gebremeskel Welegebrial |
|  | Mehari Gebre Gebremeskel Teklezgi |
| Additional Information: |  |
| Question | Response |
| Financial Disclosure  Enter a financial disclosure statement that describes the sources of funding for the work included in this submission. Review the [submission guidelines](http://journals.plos.org/plosone/s/submission-guidelines#loc-financial-disclosure-statement) for detailed | The author(s) received no specific funding for this work. |

| requirements. View published research articles from [*PLOS ONE*](https://journals.plos.org/plosone/) for specific examples.  This statement is required for submission and will appear in the published article if the submission is accepted. Please make sure it is accurate. |  |
| --- | --- |
| Funded studies  Enter a statement with the following details:   - Initials of the authors who received each award - Grant numbers awarded to each author - The full name of each funder - URL of each funder website - Did the sponsors or funders play any role in the study design, data collection and analysis, decision to publish, or preparation of the manuscript? |  |
| Did you receive funding for this work? |  |
| Competing Interests  Use the instructions below to enter a competing interest statement for this submission. On behalf of all authors, disclose any [competing interests](http://journals.plos.org/plosone/s/competing-interests) that could be perceived to bias this work—acknowledging all financial support and any other relevant financial or non- financial competing interests.  This statement is required for submission and will appear in the published article if the submission is accepted. Please make sure it is accurate and that any funding sources listed in your Funding Information later in the submission form are also declared in your Financial Disclosure statement.  View published research articles from  [*PLOS ONE*](http://journals.plos.org/plosone/) for specific examples. | The authors have declared that no competing interests exist. |

| NO authors have competing interests  Enter: *The authors have declared that no competing interests exist.*  Authors with competing interests  Enter competing interest details beginning with this statement:  *I have read the journal's policy and the authors of this manuscript have the following competing interests: [insert competing interests here]* |  |
| --- | --- |
| * typeset |  |
| Ethics Statement  Enter an ethics statement for this submission. This statement is required if the study involved:   - Human participants - Human specimens or tissue - Vertebrate animals or cephalopods - Vertebrate embryos or tissues - Field research   Write "N/A" if the submission does not require an ethics statement.  General guidance is provided below. Consult the [submission guidelines](http://journals.plos.org/plosone/s/submission-guidelines#loc-guidelines-for-specific-study-types) for detailed instructions. Make sure that all information entered here is included in the Methods section of the manuscript. | Since the investigation was a retrospective study design and secondary data analysis of openly available survey dataset from the MEASURE DHS program, ethical approval and participant consent were not necessary. The author requested DHS Program and permission was approved to download and use the dataset from: [http://www.dhsprogram.com.](http://www.dhsprogram.com/) There are no names of respondents’ addresses in the dataset files. |

| Format for specific study types  Human Subject Research (involving human participants and/or tissue)   - Give the name of the institutional review board or ethics committee that approved the study - Include the approval number and/or a statement indicating approval of this research - Indicate the form of consent obtained (written/oral) or the reason that consent was not obtained (e.g. the data were analyzed anonymously)   Animal Research (involving vertebrate animals, embryos or tissues)   - Provide the name of the Institutional Animal Care and Use Committee (IACUC) or other relevant ethics board that reviewed the study protocol, and indicate whether they approved this research or granted a formal waiver of ethical approval - Include an approval number if one was obtained - If the study involved *non-human primates*, add *additional details* about animal welfare and steps taken to ameliorate suffering - If anesthesia, euthanasia, or any kind of animal sacrifice is part of the study, include briefly which substances and/or methods were applied   Field Research  Include the following details if this study involves the collection of plant, animal, or other materials from a natural setting:   - Field permit number - Name of the institution or relevant body that granted permission |  |
| --- | --- |
|  |  |
| Data Availability  Authors are required to make all data underlying the findings described fully available, without restriction, and from the time of publication. PLOS allows rare exceptions to address legal and ethical concerns. See the [PLOS Data Policy](http://journals.plos.org/plosone/s/data-availability) and [FAQ](http://journals.plos.org/plosone/s/data-availability#loc-faqs-for-data-policy) for detailed information. | Yes - all data are fully available without restriction |

| A Data Availability Statement describing where the data can be found is required at submission. Your answers to this question constitute the Data Availability Statement and will be published in the article, if accepted. |  |
| --- | --- |
| Important: Stating ‘data available on request from the author’ is not sufficient. If your data are only available upon request, select ‘No’ for the first question and explain your exceptional situation in the text box. |  |
| Do the authors confirm that all data underlying the findings described in their manuscript are fully available without restriction? |  |
| Describe where the data may be found in full sentences. If you are copying our sample text, replace any instances of XXX with the appropriate details.   - If the data are held or will be held in a public repository, include URLs, accession numbers or DOIs. If this information will only be available after acceptance, indicate this by ticking the box below. For example: *All XXX files are available from the XXX database (accession number(s) XXX, XXX.*). - If the data are all contained within the manuscript and/or Supporting Information files, enter the following: *All relevant data are within the manuscript and its Supporting Information files.* - If neither of these applies but you are able to provide details of access elsewhere, with or without limitations, please do so. For example:   *Data cannot be shared publicly because of [XXX]. Data are available from the XXX Institutional Data Access / Ethics Committee (contact via XXX) for researchers who meet the criteria for access to confidential data.*  *The data underlying the results presented in the study are available from (include the name of the third party* | The dataset is available online and any one can access it from: [www.measuredhs.com.](http://www.measuredhs.com/) |

| *and contact information or URL).*   - This text is appropriate if the data are owned by a third party and authors do not have permission to share the data.   * typeset |  |
| --- | --- |
| Additional data availability information: |  |

<https://www2.cloud.editorialmanager.com/pone/download.aspx?id=36978254&guid=91dc9ec2-0587-4355-a373-287877d7be90&scheme=1>Manuscript	[Click here to](https://www2.cloud.editorialmanager.com/pone/download.aspx?id=36978254&guid=91dc9ec2-0587-4355-a373-287877d7be90&scheme=1)

[access/download;Manuscript;Manuscript_pediatric_2.docx](https://www2.cloud.editorialmanager.com/pone/download.aspx?id=36978254&guid=91dc9ec2-0587-4355-a373-287877d7be90&scheme=1)

# 1 Predictors of neonatal mortality among neonates in Tigray regional state, Ethiopia: a

2 **retrospective cross-sectional study**
3 Gebru Gebremeskel Gebrerufael^1^, Brhane Gebrehiwot Welegebrial^2^ and Mehari Gebre
4 Teklezgi^1^
5 ^1^Department of Statistics, College of Natural Science, Adigrat University, Adigrat, Ethiopia
6 ^2^Department of Pharmacy, College of Medicine and Health Sciences, Adigrat University,
7 Adigrat, Ethiopia

# 8 Email address:

9 **GGG**: [gebrugebremeskel2015@gmail.com](mailto:gebrugebremeskel2015@gmail.com) **ǀ ORID**: <https://orcid.org/0000-0003-2795-8529>
10 (Corresponding author)
11 **BGW**: [brhanegb008@gmail.com](mailto:brhanegb008@gmail.com) **ǀ ORCID**: <https://orcid.org/0000-0003-3600-7477>
12 **MGT:** [meharistat@gmail.com](mailto:meharistat@gmail.com) **ǀ ORCID**: <https://orcid.org/0000-0002-3638-6086>
13 ***Abstract***
14 ***Background:*** *Since 2015, Ethiopia is committed to lowering the death rate for children under*
15 *five and it is one of the countries in Sub-Saharan Africa that has accomplished the fourth*
16 *Millennium Development Goal. However, in Ethiopia, neonatal death has remained a serious*
17 *public health concern, with greater rates found in Tigray regional state and the predictors*
18 *aren’t well recognized. The goal of this study was to ascertain the prevalence of neonatal*
19 *death in the Tigray regional State as well as any relevant risk factors.*
20 ***Methods:*** *This study performed a secondary data analysis of the 2016 Ethiopia Demographic*
21 *and Health Survey (EDHS) report. Information was gathered on 716 neonates who were born*
22 *five years before the survey began. Risk factors for neonatal mortality were thought to*
23 *include mother and neonate demographics, health, and environmental factors. The study*
24 *employed multivariable logistic regression model analysis and descriptive statistics to*
25 *identify significant correlates of neonatal mortality.*
26 ***Results:*** *In Tigray regional state, the overall prevalence of neonatal mortality was 4.3%*
27 *(95% CI: 3.06, 6.10). The multivariable logistic regression model analysis revealed that*
28 *factors such as multiple birth types (AOR = 15.3, 95% CI: 3.54, 65.84), birth order (2–4)*
29 *(AOR = 4.88, 95% CI: 1.52, 15.7), sex of the neonate (being male) (AOR = 3.75, 95% CI:*
30 *1.45, 9.75), home place of delivery (AOR = 7.4, 95% CI: 2.0, 27.6), and neonates born to*
31 *the mother’s age (20–34 years) (AOR = 0.23, 95% CI: 0.087, 0.58) were significantly risk*
32 *factors associated with a higher risk of neonatal mortality rate.*
33 ***Conclusions:*** *The study recognized the sex of the neonate, birth order, mother’s age, place of*
34 *delivery, and birth type as potential risk factors for neonatal mortality. The prevalence of*
35 *neonatal mortality indicated that Tigray regional state was experiencing a higher neonatal*
36 *mortality rate than the national level. To improve services and strengthen the capacity of*
37 *local stakeholders to address the primary predictor factors that impact neonatal mortality in*
38 *the nation, the Tigray regional state has to make corrections to its public health strategy and*
39 *design.*
40 ***Keywords:*** *Neonates, Neonatal mortality, Tigray Region State, Logistic regression model*

# 41 Background

42 The probability that a neonate would die within the first 28 days of life, or the first four
43 weeks of life, is known as the neonatal mortality rate (NMR), which is calculated as the
44 number of neonate fatalities per 1,000 live births overall [**1**]. Neonatal care lasts for 28
45 complete days after delivery, starting at birth. At this point, a newborn's chances of survival
46 are at their lowest [**2**]. Every year, 2.9 million newborns worldwide pass away in their first
47 month of life; in 2014, the majority of these deaths happened in developing countries [**3**]. In
48 developing countries, the survival rate of preterm infants is extremely low [**3**]. The neonatal
49 mortality rate (NMR) accounts for about 44% of deaths in children under five, and
50 impoverished countries account for more than 99% of these deaths [**1, 4–5**]. Global public
51 health continues to be greatly concerned about neonatal mortality [**2–4**].
52 Furthermore, Sub-Saharan Africa (SSA), which has the highest rate of neonatal mortality
53 worldwide and the least success in lowering infant mortality, is where this trend is most
54 pronounced [**6**].
55 Moreover, gaining insight into the root causes of infant deaths is essential to making
56 substantial strides in public health and reducing the rates of neonatal and maternal mortality.
57 This can help as, policymakers and designers have developed suggestions and addressed
58 these linked predictor factors with appropriate interventions in order to enhance the public
59 health status of mothers and newborns. The creation and preparation of pertinent guidelines
60 and policies will safeguard the sustainability of achieving a decrease in the early infant
61 mortality rate [**7**]. Ethiopia is ranked sixth internationally and second in the SSA after
62 Nigeria, making it one of the countries with the highest NMR contributions [**8**].
63 Among the most significant predictors of neonatal mortality are the sex of the neonate [9],
64 antenatal care (ANC) follow-up [**10–11**], birth weight (low birth weight) [**10–11**], preterm
65 birth, fetal growth restriction, and congenital abnormalities [**12**]. Therefore, to lower a child's
66 high NMR, the incidence of issues during the initial neonatal period, and to ensure the
67 survival of neonatal babies, professional delivery support, excellent antenatal care, and
68 postnatal care follow-up should be implemented.
69 Over the 15 years prior, there has been a steady decline in Ethiopia's baby and under-five
70 death rates. However since 2011, NMR has raised from 29 live births per 1000 to 37 live
71 births per 1000. As a result, this report shows that it has continued to perform at a high level.
72 The 2016 Ethiopia Demographic and Health Surveys (EDHS) [**13–14**] show that the NMR of
73 34 fatalities per 1000 births in the Tigray National Regional State is higher than the national
74 average of 30 deaths per 1000 pregnancies.
75 As of 2015, Ethiopia was among the SSA countries that had achieved the fourth Millennium
76 Development Goal (MDG4), which aims to lower the mortality rate of children under five.
77 Despite a notable decline in the under-five child mortality rate, the nation's NMR remains
78 high. Thus, in order to continue working toward this goal in the future, a considerable drop in
79 NMR is necessary. Previous research indicates that during the last thirty years, early NMR
80 has declined more slowly than late NMR.
81 Interventions to lower neonatal mortality are a key concern and are part of improving
82 maternal public health care because it is vital to oversee the current health programs and set
83 policies on improving the current situation for mother and neonate health status. Furthermore,
84 knowledge of the risk factors associated with neonatal mortality is necessary to monitor the
85 effectiveness of intensive and evidence-based public health programs to reduce neonatal
86 fatalities [**15–16**].
87 Ethiopia has seen a large number of studies looking into potential risk factors for baby and
88 under-five child mortality. Studies that look at the NMR and the associated risk factors are,
89 however, scarce. Most of them are concentrated at the federal level. Because the results by
90 country level may not accurately reflect the situation at the regional level, planners and
91 policymakers should take this into mind, something that these studies neglect to do.
92 To address this identified gap, we carried out a thorough retrospective cross-sectional study
93 design analysis of the most recent EDHS 2016 to determine the main risk factors associated
94 with NMR in the Tigray regional state. We considered a variety of socioeconomic,
95 demographic, health, and environmental predictor factors in the Tigray Regional State,
96 Ethiopia [**13**].

# 97 Objective of the study

98 **General objective**
99 The general objective of this study is to assess the predictor factors associated with neonatal
100 mortality in the Tigray regional state, Ethiopia using EDHS 2016 dataset.

# 101 Specific objectives

102 ⮚ To determine the prevalence of mortality among neonates in the Tigray regional state.
103 ⮚ To identify risk factors associated with neonatal mortality among neonates in the Tigray
104 regional state.

# 105 Method and materials

106 **Source of data, study design and period**
107 Based on the EDHS 2016 dataset, this study employed a retrospective cross-sectional study
108 design with institutionally based secondary data analysis. The survey was conducted in
109 January and June of 2016 by the Ministry of Health (MOH), the Central Statistics Agency,
110 and the Ethiopian Public Health Institute (EPHI). Sponsorship was provided by the United
111 States Agency for International Development (USAID). The major objective of this survey
112 report is to give policymakers and designers detailed information on fertility, adult, newborn,
113 child, and maternal mortality, as well as the health status of mothers and children, nutrition,
114 and awareness of HIV/AIDS and other sexually transmitted illnesses (STDs).

# 115 Sample size determination technique and study population

116 This survey report employed a two-step sample selection study design. The country is divided
117 into two administrative cities and nine regional states. Using a probability proportionate to
118 size, 645 enumeration areas, 443 in rural and 202 in urban areas, were selected for the
119 survey's first phase. The newly created household list was methodically chosen in the second
120 stage, with 28 homes per cluster being chosen with an equal probability. The interview was
121 open to all reproductive women aged 15 to 49 who were stable members of the selected
122 homes and who had slept there for at least one night before the survey.
123 12,688 men and 16,650 households of reproductive age (15–59), as well as 15,683 women of
124 reproductive age (15–49), have all been questioned since the 2016 EDHS. Of these
125 households, 1,682 were in the regional state of Tigray. The Tigray regional state
126 recorded 4,428 live births in the five years preceding the research.
127 Next, neonates having completed records within the preceding five years were located using
128 the sample selection technique depicted in **Figure 1** [**17**]. Finally, samples containing a total
129 of 716 newborns were included, for whom comprehensive information encompassing all risk
130 factors considered were accessible.

131

132	Insert **Figure 1.**

133

# 134 Study area

135 Tigray regional state is located between 36- and 40-degrees East longitude north of Ethiopia,
136 bordering Eritrea to the north, Amhara to the south, Sudan to the west, and Afar to the east.
137 3,136,267 populations, of whom 1,542,165 were men, and 2,667,789 (85%) of them lived in
138 rural regions were found in the Tigray region state, according to the 2007 Ethiopian
139 population and housing censuses [**18**].

# 140 Variables of the study

141 **Response variable**
142 In the EDHS 2016 survey, mothers were asked to report any live baby deaths and
143 miscarriages. Neonatal deaths that take place in the first week of life and pregnancy losses
144 that happen after the end of the fourth week (death of a neonate between 1 and 28 days)
145 subsequently be used to compute the neonatal mortality. The study's response variable was
146 the neonatal mortality rate.
147 Thus, the outcome variable for the 𝑖^𝑡ℎ^ neonate is dichotomous, represented by a random
148 variable Y_𝑖_ that takes the value “1” with probability of success (neonatal death) and the value
149 “0” with probability of failure (no neonatal death), such that

150

Y_𝑖_ = {

1, if 𝑖^𝑡ℎ^ neonates had experienced neonatal mortality.

𝑡ℎ

0, if 𝑖	experienced neonates had not experienced with neonatal mortality.

# 151 Independent variable

152 The sociodemographic characters of the mother and the newborn, which are listed in **Table 1**,
153 served as the study's independent variables.

154

155	Insert **Table 1**

156

# 157 Statistical methods of data management and analysis

158 A statistical analysis was carried out using the statistical program Statistical Package for
159 Social Science (SPSS), version 26. Using descriptive statistics, such as frequency
160 distributions and percentages, the study looked at every variable. Tables and hands were also
161 used in the data-presenting process. One of the most widely used regression models in logistic
162 analysis is the binary logistic regression model. This method was used to estimate the event
163 probability for a categorical response variable with two outcomes.
164 The log odds model is linearly related to the predictors, such that

165

𝑌 =Log [ 𝜋𝑖 ]= 𝑏

+ 𝑏 𝑥

+ 𝑏 𝑥

… + 𝑏 𝑥

(1).

𝑖	1–𝜋𝑖	^0^

1 𝑖1

2 𝑖2

𝑝 𝑖𝑝

166	This was done in order to determine and assess the influence of each predictor on neonate

167

death. Where, 𝑌_𝑖_

=Log[ 𝜋𝑖 ] = is the value of the unobserved outcome variable for the i^th^

1−𝜋𝑖

168 case of experiencing the death of neonate’s.
169 π_𝑖_ = is the probability i^th^ case of experiencing death of neonate’s.
170 1-π_𝑖_ = is the probability of surviving in the previous five year of live birth neonate’s.
171 𝑏_0_ = is log odds of the intercept.
172 𝑥_𝑖_ = is the j^th^ predictor for the i^th^ birth.
173 𝑏_𝑗_ = is the j^th^ coefficient.
174 p = is the number of predictors.
175 The effect of covariate variables on newborn mortality was assessed using a multivariable
176 binary logistic regression model. Calculations were used to determine the adjusted odds ratios
177 (AOR) and their 95% confidence intervals (CI). The Hosmer-Lemeshow and Likelihood
178 Ratio Tests (LRT) were used to conclude the goodness of fit analysis.

179

# 180 Descriptive statistics

181 A total of 716 neonates were included in the study to investigate the risk factors for newborn
182 deaths throughout the five years prior to the survey. According to **Table 2**, out of the 716 live
183 births in the study sample, 31 (*4.3%* (95% CI: 3.06, 6.10) had been reported as neonatal
184 deaths. According to the study, newborn mortality rates were 43 per 1000 live births for the
185 five years prior to the survey. Table 2 displays the sociodemographic and economic
186 characteristics of mothers and newborns for the studies identified risk variables of neonatal
187 death.
188 About one-sixth of mothers’ were in the age group 20–34 (60.9%), and nearly one-twentieth
189 of mothers’ were below or equal to 19 years, and 34.2% were in other age groups of 35 and
190 above, respectively. More than one-third (33.0%) of mothers had no educational level (were
191 illiterate at all), and only one-eighth (13.0%) and nearly half (54.1%) of the mothers had
192 achieved a primary, secondary or above education level, respectively.
193 Regarding the birth type of neonates, more than half (97.3%) were singleton neonates. On the
194 other hand, when the sex of the neonate was concerned, half (50.1%) of the participants
195 reported being female neonates.
196 Table 2 shows that 96.9% of the respondents were from rural areas. Additionally, research
197 shows that almost 20% of respondents were drinking unprotected well water and that more
198 than half (52.0%) lacked toilet facilities.
199 The highest proportion of neonatal death rates occurs in mothers of age groups 35 and above,
200 19 and below, and 20–34 years, respectively. As mothers get older and younger, they
201 experience higher neonatal death rates. Neonatal death rates were highest (6.4%) among
202 mothers with no schooling at all. In a similar neonatal, moms of multiple birth types
203 experienced a greater rate of neonatal deaths compared to mothers of singletons (**Table 2**).

204

205	Insert **Table 2**

206

# 207 Predictor factors of neonatal mortality among neonates in the Tigray Regional State

208 **Binary logistic regression model analysis**
209 A binary logistic regression model analysis was used to ascertain the effect of each predictor
210 variable on newborn mortality.

# 211 Goodness of fit model analysis results

212 Here, we start by examining the Likelihood Ratio Test (LRT) and Hosmer-Lemeshow test
213 results for the overall goodness of fit model. A satisfactory fit for the model would be
214 indicated by the LRT's chi-square value of 76.012 with 14 degrees of freedom and a p-value
215 of 0.000. Accordingly, the Hosmer-Lemeshow test revealed that the model more accurately
216 predicted the observed data (chi-square value = 12.145 with 8 degrees of freedom and p-
217 values = 0.145; see **Table 3**).

# 218 Predictor factors associated with neonatal mortality among neonates

219 **Table 3** displays the results of an investigation of potential risk factors linked to neonatal
220 death using both a bivariable and a multivariable binary logistic regression model. From the
221 bivariable analyses, birth type, sex of the neonate, mother’s age, ANC follow-up, and place
222 of delivery were significant predictors of neonatal mortality in neonates.
223 After adjusting for other predictor variables, the result showed that birth type, sex of the
224 neonate, mother’s age, place of delivery, and birth order were statistically significant
225 predictors of neonatal death. However, ANC follow-up, toilet facility, mother's education
226 level, residence, and drinking water source were not statistically significant.
227 According to the results from **Table 3**, the odds of neonatal mortality were higher among
228 neonates that were multiple (AOR = 15.3, 95% CI: 3.54, 65.84) than those in singletons
229 births. Compared to female neonates, male neonates were more likely to die before
230 celebrating their first month of life, keeping all other variables constant. The odds of neonatal
231 mortality rates were 75% higher among male neonates (AOR = 3.75, 95% CI: 1.45, 9.75)
232 than females.
233 Concerning place of delivery, the odds of neonatal death rates were higher among neonates
234 with no health facility (at home) (AOR = 7.4, 95% CI: 2.0, 27.6) compared to those with a
235 health facility. **Table 3** also shows that the odds of neonatal death rates were lower among
236 neonates born to mothers whose ages were 20–34 (AOR = 0.23, 95% CI: 0.087–0.58)
237 compared to those who were older than or equal to 35 years old. However, there was no
238 significant difference in the risk of neonatal mortality among mothers whose ages were 19
239 and below. Neonatal mortality was shown to be substantially correlated with birth order. The
240 odds of neonatal death rates were about 88% higher among neonates whose birth order was
241 2–4 (AOR = 4.88, 95% CI: 1.52, 15.7) than those of the 1^st^ birth order (see **Table 3**).

242

243	Insert **Table 3.**

244

# 245 Discussion

246 Using the 2016 EDHS dataset, the current study empirically explored and determined the
247 potential risk factors that were linked to neonatal mortality in the Tigray regional state. Thus,
248 the prevalence of neonatal mortality in the Tigray regional state of Ethiopia was examined,
249 along with its related risk variables, using the binary logistic regression model analysis.
250 Neonatal mortality was 4.3% more common than average among neonates. This result was in
251 line with the research conducted in Indonesia, 5.2% [**19**], Nigeria, 4.1% [20], Burkina Faso,
252 4.6% [**21**], the Somali region of Ethiopia, Jimma Zone, 3.55% [**22**], and Ethiopia, 3.67%
253 [**23**]. However, the result of this study was lower than the study conducted at the University
254 of Gondar Comprehensive Specialized Hospital in Northwest Ethiopia, 17.3% [**8**], Dilchora
255 referral hospital in Dire Dawa, 11.4% [**24**], a sub-urban hospital in Cameron, 15.7% [**25**],
256 Wolaita Sodo Referral Hospital in southern Ethiopia, 17.3% [**26**], and Amhara Regional State
257 Referral Hospitals, Ethiopia, 18.6% [**2**]. In contrast, it was higher than the study conducted in
258 Debre Markos Referral Hospital in Northwest Ethiopia, 2.13% [**6**], China, 1.2% [**27**], rural
259 areas of Eastern Ethiopia, 2.84% [**28**], and Sudan, 3.0% [**29**]. This discrepancy may be
260 explained by variations in the study's design, settings, follow-up time, population sample
261 size, and sociodemographic of its participants.
262 Multiple births were more likely than singletons to result in neonatal mortality. Male
263 neonates had a higher neonatal death rate than female neonates when all other factors were
264 held equal. Similar earlier research conducted in the rural Umguza and Bubi districts also
265 revealed a strong relationship between a neonate's sex and newborn mortality [**2, 9, 30–31**].
266 The place of delivery was discovered to be a major risk factor for neonatal mortality,
267 ,meaning that newborns born at home had a larger risk of dying than newborns who received
268 care in health institutions. Studies conducted in the past have found that newborns born in
269 health facilities had greater access to delivery services and better healthcare services for them
270 babies [**8, 32**].
271 Additionally, **Table 3** showed that neonates born to women who were older had a noticeably
272 lower risk of neonatal mortality. This demonstrates once again how older women were less
273 likely to encounter neonatal mortality. Additionally, earlier studies have shown an inverse
274 relationship between maternal age and neonatal mortality [**2, 30**]. Birth order was a key
275 demographic risk factor that was significantly linked to neonatal mortality. The risk of
276 neonatal mortality was greater for newborns born in the second to fourth positions. This
277 result is in line with the results of the research done at Arba Minch General Hospital in
278 Ethiopia [33].
279 Finally, the trend showed that Ethiopia, a member of the SSA, had achieved the MDG4
280 objective in terms of neonatal mortality. However, in the Tigray regional state, development
281 is moving very slowly.

282

# 283 Conclusions and recommendations

284 The study recognized the sex of the neonate, birth order, mother’s age, place of delivery, and
285 birth type as potential risk factors for neonatal mortality. The prevalence of neonatal
286 mortality indicated that Tigray regional state was experiencing a higher neonatal mortality
287 rate than the national level. As a result, mothers who give birth at home and who have
288 multiple babies should receive special attention and prompt care from health care specialists.
289 To improve services and strengthen the capacity of local stakeholders to address the primary
290 predictor factors that impact neonatal mortality in the nation, the Tigray regional state has to
291 make corrections to its public health strategy and design.
292 Additionally, they ought to make people aware of community-based longitudinal and survival
293 studies, which can be used to identify additional, unmeasured risk factors for neonatal
294 mortality.

# 295 Abbreviations

296 ANC; **A**ntenatal **C**are; AOR: **A**djusted **O**dds **R**atio; CI: **C**onfidence **I**nterval; CSA: **C**entral
297 **S**tatistical **A**gency; EDHS: **E**thiopian **D**emographic **H**ealth **S**urvey; EPHI; **E**thiopian **P**ublic
298 **H**ealth **I**nstitute; MDG4: **M**illennium **D**evelopment **G**oal; MOH: **M**inistry of **H**ealth; NMR:
299 **N**eonatal **M**ortality **R**ate; LRT: **L**ikelihood	**R**atio **T**est; UNICEF: **U**nited **N**ations
300 **I**nternational **C**hildren’s **E**mergency **F**und; USAID: **U**nited **S**tates **A**gency for **I**nternational
301 **D**evelopment; SPSS: **S**tatistical **P**ackage of **S**ocial **S**cience; SSA: **S**ub-**S**aharan **A**frica;

# 302 Declarations

303 **Ethics approval and consent to participate**
304 Since the investigation was a retrospective study design and secondary data analysis of
305 openly available survey datasets from the MEASURE DHS program, ethical approval and
306 participant consent was not necessary. The author requested the DHS Program and permission
307 was approved to download and use the dataset from: [http://www.dhsprogram.com](http://www.dhsprogram.com/). There are
308 , no names of respondents’ addresses or addresses were given tofor respondents in the dataset files.

# 309 Consent for publication

310 Not applicable.

# 311 Availability of data and materials (ADM)

312 The dataset is available online and any one can access it from: [www.measuredhs.com](http://www.measuredhs.com/).

# 313 Competing interests

314 The author has declared that no competing interests exist.

# 315 Funding

316 The author received no specific funding for this investigation.

# 317 Author’s contribution

318 GGG, BGW, and MGT conceived the study, analyzed the data, drafted the manuscript and
319 reviewed the article.
320 Finally, all authors read and approved the final manuscript.

# 321 Acknowledgments

322 The author seriously acknowledges MEASURE DHS for giving way access to the EDHS
323 dataset.

324

325

326

327

328

329

330

331

332

333

334

335 **References**
336 1. Sankar MJ, Natarajan CK, Das RR, Agarwal R, Chandrasekaran A, Paul VK. When do
337 newborns die? A systematic review of timing of overall and cause-specific neonatal
338 deaths in developing countries. Journal of perinatology. 2016 May; 36(1):S1-1.
339 [https://doi.org/10.1038/jp.2016.27](https://doi.org/10.1186/s13052-018-0597-3)
340 2. Mengistu BA, Yismaw AE, Azene XN, Mihret MS (2021). Incidence and predictors of
341 neonatal mortality among neonates admitted in Amhara regional state referral hospitals,
342 Ethiopia: prospective follow up study (2020) 20:142. [https://doi.org/10.1186/s12887-020-](https://doi.org/10.1186/s12887-020-02031-x)
343 [02031-x](https://doi.org/10.1186/s12887-020-02031-x).
344 3. Sisay MM, Gelagay AA, and Yismaw AE. Survival and predictors among preterm
345 neonates admitted at University of Gondar comprehensive specialized hospital neonatal
346 intensive care unit, Northwest Ethiopia. Italian Journal of Pediatrics (2019) 45:4.
347 [https://doi.org/10.1186/s13052-018-0597-3.](https://doi.org/10.1186/s13052-018-0597-3)
348 4. Tran HT, Doyle LW, Lee KJ, Graham SM. A systematic review of the burden of neonatal
349 mortality and morbidity in the ASEAN Region. WHO South-East Asia journal of public
350 health. 2012; 1(3):239-48. <https://doi.org/10.4103/2224-3151.207020>
351 5. Mekonnen Y, Tensou B, Telake DS, Degefie T, Bekele A. Neonatal mortality in Ethiopia:
352 trends and determinants. BMC public health. 2013; 13:1-4. [https://doi.org/10.1186/1471-](https://doi.org/10.1186/1471-2458-13-483)
353 [2458-13-483](https://doi.org/10.1186/1471-2458-13-483)
354 6. Alebel A, Wagnew F, Petrucka P, Tesema C, Moges NA, Ketema DB, Yismaw L,
355 Melkamu MW, Hibstie YT, Temesgen B, Bitew ZW, Tadesse AA, and Kibret GD.
356 Neonatal mortality in the neonatal intensive care unit of Debre Markos referral hospital,
357 Northwest	Ethiopia:	a	prospective	cohort	study.	(2020)	20:72.
358 [https://doi.org/10.1186/s12887-020-1963-z.](https://doi.org/10.1186/s12887-020-1963-z)
359 7. World Health Organization. Neonatal and perinatal mortality: country, regional and
360 global estimates. Geneva: World Health Organization; 2006.
361 8. Gudayu TW, Zeleke EG, Lakew AM. Time to death and its predictors among neonates
362 admitted in the intensive care unit of the University of Gondar Comprehensive
363 Specialized Hospital, Northwest Ethiopia. Research and Reports in Neonatology. 2020
364 Jan 29:1-0. <https://doi.org/10.2147/RR.S233828>.
365 9. Kolola T, Ekubay M, Tesfa E, Morka W. Determinants of neonatal mortality in north
366 Shoa zone, Amhara regional state, Ethiopia. PloS one. 2016; 11(10):e0164472.
367 [https://doi.org/10.1371/journal.pone.0164472.](https://doi.org/10.1371/journal.pone.0164472)
368 10. Akinyemi, J.O., Bamgboye, E.A. & Ayeni, O. Trends in neonatal mortality in Nigeria and
369 effects of bio-demographic and maternal characteristics. BMC Pediatr 15, 36 (2015).
370 [https://doi.org/10.1186/s12887-015-0349-0.](https://doi.org/10.1186/s12887-015-0349-0)
371 11. Kananura, R.M., Tetui, M., Mutebi, A. et al. The neonatal mortality and its determinants
372 in	rural	communities	of	Eastern	Uganda. Reprod	Health **13**,	13	(2016).
373 [https://doi.org/10.1186/s12978-016-0119-y.](https://doi.org/10.1186/s12978-016-0119-y)
374 12. Allanson, E.R., Muller, M. & Pattinson, R.C. Causes of perinatal mortality and associated
375 maternal complications in a South African province: challenges in predicting poor
376 outcomes. BMC Pregnancy Childbirth **15**, 37 (2015). [https://doi.org/10.1186/s12884-015-](https://doi.org/10.1186/s12884-015-0472-9)
377 [0472-9.](https://doi.org/10.1186/s12884-015-0472-9)
378 13. Central Statistical Agency [Ethiopia] and ICF International. Ethiopia demographic and
379 health survey 2011. Addis Ababa, Ethiopia and Calverton, Maryland, USA: Central
380 Statistical Agency and ICF International; 2012.
381 14. Oestergaard MZ, Inoue M, Yoshida S, Mahanani WR, Gore FM, Cousens S, Lawn JE,
382 Mathers CD, United Nations Inter-Agency Group for Child Mortality Estimation and the
383 Child Health Epidemiology Reference Group. Neonatal mortality levels for 193 countries
384 in 2009 with trends since 1990: a systematic analysis of progress, projections, and
385 priorities. PLoS medicine. 2011; 8(8):e1001080.
386 15. Black RE, Cousens S, Johnson HL, Lawn JE, Rudan I, Bassani DG, Jha P, Campbell H,
387 Walker CF, Cibulskis R, Eisele T. Presentation for the Child Health Epidemiology
388 Reference Group of WHO and UNICEF. Global, regional, and national causes of child
389 mortality in 2008: A systematic analysis. The Lancet. 2010; 375(9730):1969-87.
390 <https://doi.org/10.1016/S0140-6736> (10)60549-1
391 16. Lawn JE, Lee AC, Kinney M, Sibley L, Carlo WA, Paul VK, Pattinson R, Darmstadt GL.
392 Two million intrapartum-related stillbirths and neonatal deaths: where, why, and what can
393 be done? International Journal of Gynecology & Obstetrics. 2009; 107:S5-19.
394 [https://doi.org/10.1016/j.ijgo.2009.07.016.](https://doi.org/10.1016/j.ijgo.2009.07.016)
395 17. Central Statistical Agency (CSA) [Ethiopia] and ICF. 2016. Ethiopia Demographic and
396 Health Survey 2016. Addis Ababa, Ethiopia, and Rockville, Maryland, USA: CSA and
397 ICF.
398 18. Central Statistical Agency, 2007 Census of Population.
399 19. Anggondowati, T., El-Mohandes, A.A.E., Qomariyah, S.N. et al. Maternal characteristics
400 and obstetrical complications impact neonatal outcomes in Indonesia: a prospective
401 study. BMC Pregnancy Childbirth **17**, 100 (2017). [https://doi.org/10.1186/s12884-017-](https://doi.org/10.1186/s12884-017-1280-1)
402 [1280-1.](https://doi.org/10.1186/s12884-017-1280-1)
403 20. Akinyemi, J.O., Bamgboye, E.A. & Ayeni, O. Trends in neonatal mortality in Nigeria and
404 effects of bio-demographic and maternal characteristics. BMC Pediatr **15**, 36 (2015).
405 [https://doi.org/10.1186/s12887-015-0349-0.](https://doi.org/10.1186/s12887-015-0349-0)
406 21. Diallo AH, Meda N, Ouedraogo WT, Cousens S, Tylleskar T. A prospective study on
407 neonatal mortality and its predictors in a rural area in Burkina Faso: can MDG-4 be met
408 by 2015?. Journal of Perinatology. 2011; 31(10):656-63.
409 22. Debelew GT, Afework MF, Yalew AW. Determinants and causes of neonatal mortality in
410 Jimma zone, southwest Ethiopia: a multilevel analysis of prospective follow up study.
411 PloS one. 2014; 9(9):e107184. <https://doi.org/10.1371/journal.pone.0107184>.
412 23. Mekonnen, Y., Tensou, B., Telake, D.S. et al. Neonatal mortality in Ethiopia: trends and
413 determinants. BMC Public Health **13**, 483 (2013). [https://doi.org/10.1186/1471-2458-13-](https://doi.org/10.1186/1471-2458-13-483)
414 [483](https://doi.org/10.1186/1471-2458-13-483).
415 24. Roba AA, Diro DH. Morbidities, rate and time trends of neonatal mortality in Dilchora
416 Referral Hospital, Dire Dawa, Ethiopia, 2012-2017. Austin Medical Sciences. 2017;
417 2(1):1019. [http://www.austinpublishinggroup.com](http://www.austinpublishinggroup.com/)
418 25. Ndombo, P.K., Ekei, Q.M., Tochie, J.N. et al. A cohort analysis of neonatal hospital
419 mortality rate and predictors of neonatal mortality in a sub-urban hospital of
420 Cameroon. Ital J Pediatr 43, 52 (2017). [https://doi.org/10.1186/s13052-017-0369-5.](https://doi.org/10.1186/s13052-017-0369-5)
421 26. Mihiretu A, Negash T, Elazar T. Perinatal death and associated factors in Wolaita Sodo
422 referral hospital, southern Ethiopia: a facility based cross-sectional study. Prim Health
423 Care. 2017; 7(2):1-5. <https://doi.org/10.4172/2167-1079.1000269>.
424 27. Wang CH, Du LZ, Ma XL, Shi LP, Tong XM, Liu H, Ding GF, Yi B, Pan XN, Zhong
425 DN, Liu L. Analysis of in-hospital neonatal death in the tertiary neonatal intensive care
426 unit in China: a multicenter retrospective study. Chinese medical journal. 2016;
427 129(22):2652-8.
428 28. Desta BN, Assefa N, Damte TD, Hordofa LO. Neonatal mortality and its risk factors in
429 eastern Ethiopia: a prospective cohort study in Kersa health and demographic surveillance
430 system (Kersa HDSS). Mortality. 2016; 13(18):19.
431 29. Bashir, A.O., Ibrahim, G.H., Bashier, I.A. et al. Neonatal mortality in Sudan: analysis of
432 the	Sudan	household	survey,	2010. BMC	Public	Health **13**,	287	(2013).
433 <https://doi.org/10.1186/1471-2458-13-287>.
434 30. Mbiba V. Factors associated with perinatal mortality in Umguza and Bubi rural areas,
435 2015-the effect of maternal human immunodeficiency virus status. UNSPECIFIED:
436 UNSPECIFIED thesis. 2015 Aug.
437 31. Tessema, Z.T., Tesema, G.A. Incidence of neonatal mortality and its predictors among
438 live births in Ethiopia: Gompertz gamma shared frailty model. Ital J Pediatr 46, 138
439 (2020). [https://doi.org/10.1186/s13052-020-00893-6.](https://doi.org/10.1186/s13052-020-00893-6)
440 32. Berhie, K.A., Gebresilassie, H.G. Logistic regression analysis on the determinants of
441 stillbirth	in	Ethiopia. matern	health,	neonatol	and	perinatol **2**,	10	(2016).

| 442 | [https://doi.org/10.1186/s40748-016-0038-5.](https://doi.org/10.1186/s40748-016-0038-5) |
| --- | --- |
| 443 33. | Dessu S, Kote M, Gebremeskel F, Girum T. Predictors of neonatal mortality among |
| 444 | neonates who admitted in neonatal intensive care unit at Arba Minch General Hospital. |
| 445 | Ethiopian Journal of Health Development. 2019; 33(1). |

446

447

448

449

450

451

452

453

454

455

456

457

458

459

460

461

462

# 463 Table with captions

464	**Table 1:** Operational definition and category of independent variables used in the study

Variables	Description and categories

Mother’s age	Mother’s age (35 and above, 20–34, 19 and below )

Birth type	Birth type neonate (singletons, multiple)

Sex of neonate	Sex of neonate (female, male)

ANC follow-up	ANC follow-up (no, yes)

Toilet facility	Toilet facility using of mother’s (with facility, no facility)

Mother's education level	Mother's education level (no education, primary and secondary, tertiary) Residence	Residence place of neonates (rural, urban)

Drinking water source	Drinking water source of mother's (protected, piped, unprotected) Place of delivery	Place of delivery of neonate (health facility, home)

465

466

467

468

469

470

471

472

473

474

475

476

477

478	**Table 2:** Descriptive characteristics of the respondents in the Tigray Regional State, Ethiopia,

479	2016 (n = 716).

Neonate survival status	Total	frequency

| Variables | Categories |  | Alive (%) | Died (%) | (%) |
| --- | --- | --- | --- | --- | --- |
| Mother’s age | 19 and below |  | 34 (97.1%) | 1 (2.9%) | 35 (4.9%) |
|  | 20–34 |  | 427 (97.9%) | 9 (2.1%) | 436 (60.9%) |
|  | 35 and above |  | 224 (91.4%) | 21 (8.6%) | 245 (34.2%) |
| Birth type | Multiple |  | 13 (68.4%) | 6 (31.6%) | 19 (2.7%) |
|  | Singletons |  | 672 (96.4%) | 25 (3.6%) | 697 (97.3%) |
| Sex of neonate | Male |  | 333 (93.3%) | 24 (6.7%) | 357 (49.9%) |
|  | Female |  | 352 (98.1%) | 7 (2.0%) | 359 (50.1%) |
| ANC follow-up | Yes |  | 574 (96.5%) | 21 (3.5%) | 595 (83.1%) |
|  | No |  | 111 (91.7%) | 10 (8.3%) | 121 (16.9%) |
| Toilet facility | No facility |  | 355 (95.4%) | 17 (4.6%) | 372 (52.0%) |
|  | With facility |  | 330 (95.9%) | 14 (4.1%) | 344 (48.0%) |
|  | Secondary | and | 372 (96.1%) | 15 (3.9%) | 387 (54.1%) |
| Mother's education level | above |  |  |  |  |
|  | Primary |  | 92 (98.9%) | 1 (1.1%) | 93 (13.0%) |
|  | No education |  | 221 (93.6%) | 15 (6.4%) | 236 (33.0%) |
| Residence | Urban |  | 20 (90.9%) | 2 (9.1%) | 22 (3.1%) |
|  | Rural |  | 665 (95.8%) | 29 (4.2%) | 694 (96.9%) |
| Drinking water source | Unprotected |  | 131 (97%) | 4 (3.0%) | 135 (18.9%) |
|  | Piped |  | 170 (95%) | 9 (5.0%) | 179 (25%) |
|  | Protected |  | 384 (95.5%) | 18 (4.5%) | 402 (56.1%) |
| Place of delivery | Health facility |  | 368 (92.9%) | 28 (7.1%) | 396 (55.3%) |
|  | Home |  | 317 (99.1%) | 3 (0.94%) | 320 (44.7%) |

Total number of neonates under one month	685 (95.7%)	31 (4.3%)	716 (100%)

480

481	**Table 3:** The bi-variable and multivariable binary logistic regression analysis of predictor

482	factors associated with neonatal mortality among neonates in the Tigray Regional State, 2016

483	(n = 716).

Bi-variable	binary

logistic	Multivariable binary logistic

COR	(95%	CI	for

| Variables |  | COR) | AOR (95% CI for AOR) |
| --- | --- | --- | --- |
| **Mother’s age (ref.= 35 and above )** |  | 1 | 1 |
| 19 and below |  | 0.314 (0.041, 2.41) | 0.263 (0.026, 2.692) |
| 20-34 |  | 0.225 (0.101, 0.499)* | 0.23 (0.087, 0.58)* |
| **Birth type (ref.= singletons )**  Multiple |  | 1  12.4 (4.4, 35.3)* | 1  15.3 (3.54, 65.84)* |
| **Sex of neonate (ref.= female)**  Male |  | 1  3.6 (1.54, 8.52)* | 1  3.75 (1.45, 9.75)* |
| ANC follow-up (ref.= no ) |  | 1 | 1 |
| Yes |  | 0.41 (0.19, 0.890)* | 0.20 (0.025, 1.63) |
| Toilet facility (ref.= with facility ) |  | 1 | 1 |
| No facility |  | 1.13 (0.55, 2.33) | 2.06 (0.613, 6.95) |
| Mother's education level (ref.= | no |  |  |
| education ) |  | 1 | 1 |
| Secondary and above |  | 0.60 (0.29, 1.24) | 0.46 (0.125, 1.67) |
| Primary |  | 0.20 (0.021, 1.23) | 0.22 (0.024, 2.06) |
| Residence (ref.= rural) |  | 1 | 1 |

| Urban | 2.3 (0.51, 10.3) | 1.11 (0.085, 14.37) |
| --- | --- | --- |
| Drinking water source (ref.= protected ) | 1 | 1 |
| Unprotected | 0.65 (0.22, 1.96) | 0.54 (0.16, 1.81) |
| piped | 1.13 (0.50, 2.57) | 0.35 (0.048, 2.59) |
| **Place of delivery (ref.= health facility)** | 1 | 1 |
| Home | 8.0 (2.42, 26.7)* | 7.4 (2.0, 27.6)* |
| **Birth order (ref.= 1st )** | 1 | 1 |
| 2–4 | 2.14 (0.83, 5.60) | 4.88 (1.52, 15.7)* |
| 5 and above | 1.17 (0.45, 3.03) | 1.10 (0.335, 3.32) |

* indicates significant predictors which have p-value <0.05, ANC: Antenatal Care,

**NB**: LRT = 76.012 (p < 0.000),

Hosmer-Lemeshow Test = 12.145 (p-value = 0.145)

484

485

486

487

488

489

490

491

492

493

494

495

# 496 Figures with captions

497	**Figure 1:** Diagrammatic presentation of sample selection among neonates included in this

498	study, 2016
